# Accelerating vaccine research and development for skin neglected tropical diseases: A case for leishmaniasis, leprosy, and Buruli ulcer

**DOI:** 10.1371/journal.pntd.0014375

**Published:** 2026-06-05

**Authors:** Hua Wang, Fernanda Oliveira Novais, Maria Adelaida Gómez, Stephen Muhi, Camila I. de Oliveira, Thao-Thy Pham, Samantha Vermaak, VALIDATE Network Skin NTDs Working Group, Rajko Reljic

**Affiliations:** 1 Strathclyde Institute of Pharmacy & Biomedical Sciences, University of Strathclyde, Glasgow, United Kingdom; 2 Department of Microbial Infection and Immunity, College of Medicine, The Ohio State University, Columbus, Ohio, United States of America; 3 Centro Internacional de Entrenamiento e Investigaciones Médicas, Cali, Colombia‌‌; 4 Departamento de Ciencias Biológicas, Universidad Icesi, Cali, Colombia; 5 Department of Microbiology and Immunology, Doherty Institute for Infection and Immunity, The University of Melbourne, Melbourne, Australia; 6 Victorian Infectious Diseases Service, Royal Melbourne Hospital, Parkville, Victoria, Australia‌‌; 7 Fundação Oswaldo Cruz, Fiocruz, Instituto Gonçalo Muniz, Salvador, Brazil; 8 Instituto Nacional de Ciência e Tecnologia em Doenças Tropicais, Salvador, Bahia, Brazil; 9 Clinical Immunology Unit, Department of Clinical Sciences, Institute of Tropical Medicine, Antwerp, Belgium; 10 The Jenner Institute, University of Oxford, Oxford, United Kingdom; 11 Institute for Infection and Immunity, City St. George’s University of London, London, United Kingdom; Wadsworth Center, New York State, UNITED STATES OF AMERICA

## Abstract

Neglected tropical diseases (NTDs), particularly those with prominent cutaneous manifestations such as leishmaniasis, leprosy, and Buruli ulcer, represent a substantial global health burden, affecting hundreds of millions of people and perpetuating cycles of poverty and disability. Despite the current availability of treatment strategies, vaccines remain the most sustainable and cost-effective intervention that can reduce reliance on chemotherapeutics. However, vaccine research and development (R&D) for these diseases face considerable challenges that cannot be overcome without a strategic shift in response by national and international health programmes and organisations, research funders, and the pharmaceutical industry. This paper draws on collective insights from the VALIDATE Network workshop on “Vaccines for Skin Neglected Tropical Diseases—Progress and Challenges” (Bogotá, Colombia, 5–8 May 2025). We advocate for a multisectoral shift across three critical pillars: i) an increase in funding for NTD vaccine R&D, ii) integration of NTD vaccine R&D into the preparedness and response policies by international agencies and local governments, and iii) fostering patient and public engagement and advocacy for NTD vaccine R&D and implementation. Coordinated efforts across these three pillars will unlock the transformative potential of vaccines and substantially reduce the health, societal, and economic burdens from these diseases.

## Introduction‌‌

Neglected tropical diseases (NTDs) represent a varied collection of illnesses predominantly found in tropical and subtropical areas, deeply entrenched within impoverished communities [1,2]. These conditions cause roughly 120,000 deaths annually and are fundamentally tied to global poverty and inequality, which in turn amplify poor health, restrict educational access, and diminish worker output. Furthermore, NTDs result in an estimated 14.1 million disability-adjusted life years (DALYs) lost each year, creating a massive economic burden through billions of dollars in direct healthcare expenses and lost productivity across developing nations [3].

Nearly half of NTDs defined by the World Health Organization (WHO) manifest primarily in the skin [1,2]. This paper focuses on skin NTDs caused by the intracellular pathogens *Leishmania* (cutaneous, mucocutaneous, and post-kala azar dermal leishmaniasis) and *Mycobacteria* (leprosy and Buruli ulcer, BU), which exemplify the societal and scientific challenges posed by NTDs, as they lead to chronic disability, severe disfigurement, and profound social stigma [4]. More broadly, the catastrophic health expenditure, the proportion of direct and indirect costs of NTD diagnosis and treatment relative to household income, can exceed 11% and reach up to 92% for patients, often pushing families into financial hardship and debt [5]. This financial strain contributes to high rates of under-reporting, reduced adherence to prolonged therapeutic schemes, and perpetuation of transmission cycles, substantially increasing costs to the public health sector [6,7]. This interconnectedness means that investment in NTD vaccines is not merely a health intervention, but a fundamental strategy for economic development and social equity. Yet, vaccines remain largely absent from the global health arsenal, with no approved human vaccines specifically targeting leishmaniasis, leprosy, and BU.

The research and development (R&D) of effective, safe, affordable, and accessible NTD vaccines is crucial to break the self-perpetuating cycle of poverty and disease. This is especially relevant where existing drugs face challenges such as severe toxicity, increasing resistance, accessibility constraints, and poor affordability, as is the case for antileishmanial and antimycobacterial drugs. Even so, major hurdles limit skin NTD vaccine R&D: 1) the biological complexity of the host-pathogen relationship especially for intracellular pathogens; 2) insufficient interest and support from funders and pharmaceutical companies; 3) epidemiological gaps, such as limited understanding of disease burden; 4) insufficient cost-of-illness data to justify large-scale investment; and 5) policy gaps, such as the lack of mechanisms to accelerate clinical trials and regulatory approvals (similar to those that operated during the COVID-19 pandemic) to help reduce delays and align international agencies and local governments with the health urgency. These are further compounded by emerging global challenges, including climate change, migratory crises, and shifting societal perceptions of the risks posed by infectious diseases that altogether threaten coordinated response efforts [8–10].

**Fig 1:**
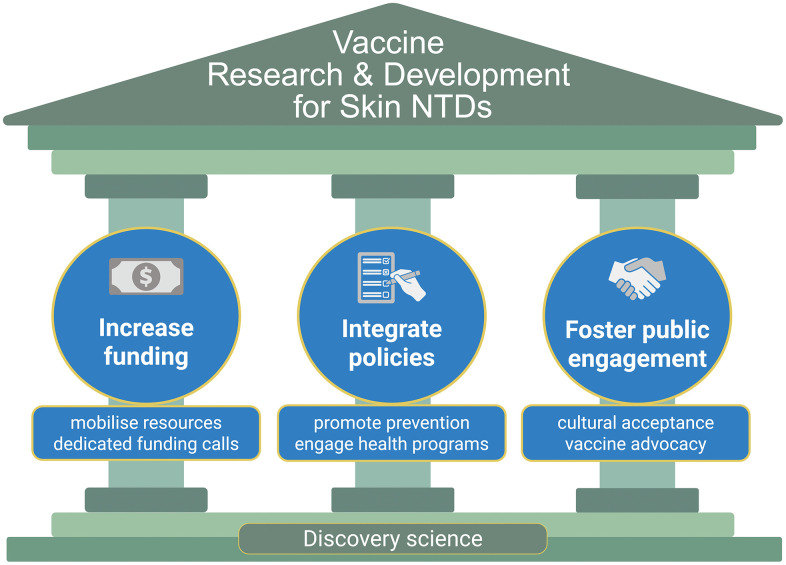
Pillars for advancing vaccines against skin NTDs. Success in vaccine development requires: (1) increased and dedicated funding for research and development; (2) integration of policies that prioritise prevention and strengthen health programs; and (3) enhanced patient and public engagement and vaccine advocacy. At the foundation of these efforts lies strong discovery science, which drives all subsequent progress. Created in BioRender. Novais, F. (2026) https://BioRender.com/7tfd1o9.

VALIDATE (www.validate-network.org) is an international research network connecting >900 members in 80 countries working towards the development of efficacious vaccines against three groups of NTDs (*Mycobacterium* spp., *Leishmania* spp., and *Burkholderia pseudomallei*). In alignment with WHO’s vision of integrated management and control of skin NTDs, VALIDATE conducted a 3-day workshop on “Vaccines for Skin Neglected Tropical Diseases—Progress and Challenges” with presentations and roundtable discussions involving 29 delegates from 11 countries (5 Low- and Middle-Income Countries, LMICs), representing different sectors (academia, research, funding bodies, charities, nongovernmental organisations, and government officials). Results from this workshop led us to propose an integrated approach for skin NTD vaccine R&D across three critical pillars to address the health, societal, and financial challenges brought on by these diseases (Fig 1): i) increase in funding, ii) integration into preparedness and response policies by international agencies and local governments, and iii) fostering patient and public engagement and advocacy.

## Current progress in skin NTD vaccine R&D and challenges

### Leishmaniasis

The leishmaniases represent a spectrum of clinical diseases, caused by protozoan parasites of the genus *Leishmania*, that are endemic in >90 countries spread over Africa, America, Asia, and Europe [11]. Disease manifestations can range from primary localised cutaneous ulcers (cutaneous leishmaniasis, CL) to debilitating mucosal infections and fatal visceral leishmaniasis (VL; kala-azar). Globally, CL accounts for an estimated 0.7–1 million new cases annually [12]. In regions of VL elimination efforts, the prevalence of post-kala azar dermal leishmaniasis (disseminated nodular, papular, or macular lesions secondary to drug-dependent healing of VL) has increased. This, in addition to the high rates of under-reporting due to stigma and geographic and public healthcare access barriers, indicates that the burden of CL may be higher than anticipated [13,14].

The available control strategies for leishmaniasis are vector management (in areas without sylvatic transmission) and active case detection followed by treatment, both with substantial implementation barriers [12]. Traditional first-line chemotherapeutic treatment relies on toxic parenteral drugs with prolonged therapeutic schemes (>20 days), which are increasingly challenged by drug resistance, limited adherence, and barriers to access, threatening this control measure. Local treatments (such as thermotherapy, cryotherapy, or intralesional antimony) are alternatives for patients presenting uncomplicated CL, however only representing 20% to 50% of cases in some endemic areas [15]. Thus, a vaccine is urgently needed. Modelling studies indicate that a vaccine with even modest efficacy (50%) and short-term protection (5 years) would be more cost-effective than existing chemotherapies for CL and VL (*e.g.*, a two-dose vaccine, $0.5/dose, compared to $180 for a 20-day regimen of pentavalent antimonial drugs) [16].

The potential for vaccination against leishmaniasis is strongly supported by extensive experimental data and epidemiological evidence of long-term immunity after primary natural infection, manifested by low recurrence rates in endemic areas [17–19]. This is further supported by the historical practice of leishmanisation, a controlled infection with low virulence live *Leishmania* promastigotes (*L. major),* employed as a formal vaccination programme in the Middle East but discontinued in the 90s due to safety concerns from nonhealing lesions in some cases [20].

Globally, CL can be caused by more than 20 different *Leishmania* species, questioning the feasibility of one vaccine offering cross-species protection [12]. However, genome sequencing revealed >99% of genes with conserved synteny between the evolutionary divergent *L. infantum, L. braziliensis,* and *L. major* species, and high conservation in the coding sequences with amino acid identity >80%, suggesting the potential for cross-protection [21,22]. Importantly, clinical observations suggest that immunity acquired through infection with one *Leishmania* species can provide a degree of cross-protection against others, raising the possibility that vaccines designed for CL could also confer protection against the more severe VL, and vice versa [23–25]. However, challenges for universal vaccine development may arise from the high genome plasticity evidenced in *Leishmania,* potentially impacting antigen conservation and immunodominance [26].

Several leishmaniasis vaccine candidates (LEISH-F1, LEISH-F2, LEISH-F3, and ChAd63-KH) have been evaluated in clinical trials for safety and immunogenicity in India, the US, the UK, Sudan, and Peru [17,27–30]. Recombinant subunit protein vaccines LEISH-F1, F2, and F3 have demonstrated safety and immunogenicity (NCT00111514, NCT00982774, NCT01484548, and NCT02071758), and the ability to induce Th1-mediated cellular immune response characterised by IFN-γ, TNF-α, and IL-2 production to activate macrophage-mediated killing of intracellular *Leishmania* parasites. Notably, LEISH F2 completed Phase II study (NCT01011309) showing therapeutic effects in CL patients [30–33]. ChAd63-KH, a replication-deficient adenovirus expressing a novel synthetic gene encoding two leishmanial proteins KMP-11 and HASPB, has progressed through a human trial in healthy UK volunteers [34]. Unfortunately, while Phase IIa study showed ChAd63-KH to be safe and immunogenic, a randomised, double-blind Phase IIb trial found no significant therapeutic benefit, with no improvement of skin lesions compared to the placebo, among Sudanese patients with post kala-azar dermal leishmaniasis [29,35]. Of candidates currently positioned for future trials, LmCen^−/−^, a CRISPR-generated live-attenuated *L. major* strain, has demonstrated 82.5% efficacy against canine VL and Phase I human trials are planned, alongside Leish F1F3, a recombinant chimeric protein vaccine designed to promote early Th1-mediated cellular and regulatory immune responses, has demonstrated cross-species protection and strong intradermal response to leishmanial antigen in murine infection models [36–38].

The progression of the vaccine candidates to Phase III is hindered by the poor translatability of pre-clinical models and the absence of well-defined correlates of protection, which complicates both rational vaccine design and the determination of feasible trial sample sizes. Although murine models clearly dichotomise Th1 and Th2 responses mediating resistance and susceptibility to infection, respectively, this is not the case for all human dermal leishmaniases [39–42]. Ulcerated chronic lesions and mucocutaneous leishmaniasis illustrate this, where exacerbated Th1 responses contribute to sustained pathology [43,44]. Achieving effective, durable protection with an effective vaccine requires the induction of finely controlled immune responses, further shaped by optimal antigen-adjuvant-delivery combinations, an understudied area in the field of leishmaniasis vaccinology. Progress is accelerating; high-throughput approaches such as immunopeptidomics enable direct identification of *Leishmania*-derived antigens presented in patients, ensuring relevance to natural human infections [45,46]. In addition, a controlled human infection model of cutaneous leishmaniasis using natural sand fly transmission has been established, allowing early human efficacy signals and in-depth immunological profiling already in Phase I stage [47].

The WHO has set ambitious 2030 targets for leishmaniasis, including eliminating VL as a public health problem and detection of 85% of global CL cases, with 95% of these receiving treatment [2,48]. Despite this, global and national strategies still omit human vaccine development, leaving these efficient and preventive tools underfunded, creating a barrier to sustainably reduce the burden of VL and CL.

### Leprosy

Leprosy or Hansen’s disease is one of the oldest known human diseases. It is a chronic and stigmatised infectious disease caused by *Mycobacterium leprae* and *M. lepromatosis* bacteria. It primarily affects the skin, peripheral nerves, upper respiratory tract, and eyes. Leprosy is endemic in regions of the Americas, Africa, and South-East Asia. Worldwide, there are over 200,000 new cases every year, but that number is likely an underestimate, as people affected by leprosy tend to avoid surveillance systems [49].

Leprosy is curable with multi-drug therapy (MDT), which consists of three drugs (dapsone, rifampicin, and clofazimine), but early diagnosis and treatment are critical. Late diagnosis or if left untreated, leaves patients at risk of permanent nerve damage that can result in the loss of sensation, paralysis, and deformity that lead to permanent disability and stigma. Based on skin lesions and bacillary load from skin slit smear test, the MDT regimen is either 6 monthly doses of rifampicin for paucibacillary leprosy (for 1–5 skin lesions with no bacilli count), and 12 monthly doses for multibacillary leprosy (with 6 or more lesions or nerve involvement and/or positive bacilli count), combined with daily doses of dapsone and clofazimine [49]. MDT has significantly decreased the disease burden over the years, but relapse can occur and leprosy is yet to be eliminated, with a considerable burden of disability due to late diagnosis and on-going transmission, particularly evident in paediatric cases [50]. The “elimination” declaration by WHO is defined as “the reduction of prevalence to a level below one case per 10,000 population”. This target may not go far enough for a disease as stigmatised as leprosy and may unduly shift resources and financial support needed for patient care and research [51–53].

Prevention strategies for leprosy are challenging because the transmission mechanism is not exactly clear, the bacilli cultivation to study infection requires animals (mice and nine-banded armadillos), and the disease has a long incubation period, from 6 months to 20 years before symptoms manifest in a clinically diagnosable form. The WHO recommends contact tracing and a single dose of rifampicin as preventive chemotherapy for anyone in close contact with a leprosy patient. However, the use of antibiotics to prevent leprosy is not a long-term solution and can exacerbate the rise of antibiotic resistance, risking treatment options for other mycobacterial infections, making MDT futile, and adding to the already major challenge that *M. leprae* and *M. lepromatosis* cannot be easily cultivated in the lab for either diagnostic or research purposes [54].

No approved leprosy-specific vaccine currently exists, though BCG vaccine (attenuated *M. bovis* used against tuberculosis since 1921) offers variable protection. In 2018, the WHO recommended BCG be given to healthy neonates at birth for leprosy prevention [55]. However, clinical trials provide conflicting data, with an efficacy range between 18 and 90%, and demonstrable waning protection within 10 years [56–59].

Several leprosy vaccine candidates have emerged over the years, but the majority have not progressed beyond pre-clinical and clinical stages [60,61]. The developments involved the formulation of bacilli of the same genus as *M. leprae*, such as *M. habana*, *M. vaccae*, ICRC (Indian Cancer Research Centre) bacilli, and MIP (*Mycobacterium indicus pranii,* formerly Mw). These were tested in clinical trials for disease prevention in exposed populations. None demonstrated overall superior efficacy compared to BCG. However, MIP did demonstrate protective efficacy of 68%, 59%, and 39% at 3-, 6-, and 9-year follow-up, respectively [62]. Interestingly, MIP has also proven to be efficient as an adjunct to MDT, improving clinical endpoints in patients (*e.g.*, quicker clinical improvements, clearance of granulomas, and a drop in bacilli count) [63]. MIP is approved by the Drugs Controller General of India and the U.S. Food and Drug Administration (FDA), and it has been licensed to Cadila Pharmaceuticals. In 2017, the Indian Council for Medical Research launched an immunisation project to eradicate leprosy in leprosy-endemic districts using MIP in addition to MDT [64]. Furthermore, there is an on-going phase III randomised controlled study in India (CTRI/2021/09/036335) to evaluate and compare the immunotherapeutic efficacy of MIP and BCG-vaccines as an adjunct to chemotherapy in multibacillary leprosy patients.

There is a promising new vaccine candidate based on defined antigens and human immunology: LepVax [65]. It consists of two active ingredients, the Lep-F1 antigen, a tetravalent fusion protein engineered with three *M. leprae* T cell antigens and a protein expression stabiliser, and the adjuvant, a synthetic Toll-like receptor 4 agonist glucopyranosyl lipid A in a stable emulsion that promotes antigen-specific Th1 responses. It had a successful Phase I safety and immunogenicity trial in healthy volunteers in the U.S. under FDA regulation, and is now in Phase Ib/IIa clinical trial to assess its safety and efficacy in leprosy-endemic regions in Brazil [66].

As is the case for leishmaniasis, advancement of clinical trials for leprosy vaccines requires a better understanding of the immune mechanisms of protection against *M. leprae*, identifying correlates of protection, determining efficacy endpoints for vaccine trials, ensuring safety and effectiveness in people with subclinical *M. leprae* infection, and having long-term financial support and investment. Furthermore, social and sometimes institutionalised stigma associated with leprosy can be a significant barrier to care-seeking and participation in health interventions. For example, there are 127 discriminatory laws in 22 countries (most in India) based on leprosy [67]. These laws (*e.g.*, employment opportunities, segregation, divorce, and disqualification to stand in an election) are often relics of outdated, colonial-era fears and misconceptions about the disease, primarily stemming from the false belief that it is highly contagious.

The WHO Global Leprosy Strategy 2021–2030 is structured around four key priorities: implementing integrated, country-owned zero leprosy roadmaps; scaling up leprosy prevention alongside integrated active case detection; managing leprosy and its complications to prevent new disability; and combating stigma while ensuring human rights [67]. Vaccine is part of strategic objectives for integration and prevention with existing and potential new vaccines, but vaccine R&D is not explicitly mentioned as a priority or a dedicated goal. This strategic oversight hinders a truly proactive elimination approach, as a vaccine is essential for interrupting transmission and achieving the long-term goal of elimination of the disease as a health threat.

### Buruli ulcer (BU)

BUs, caused by *M. ulcerans*, are typically slowly progressive lesions characterised by extensive tissue destruction. Delayed treatment can result in cosmetic deformity, functional impairment, disability, and psychological harm, all of which are intrinsically linked to poverty and inequity [68,69]. Its profound impact on public health necessitates a strategic shift towards preventive interventions, notably vaccination. BU is reported in over 30 countries, predominantly in West Africa and Australia [68]. While global reported cases to the WHO have decreased, this trend masks the rapidly increasing incidence in Australia and the challenges of reporting cases in stigmatised and vulnerable African communities [70]. In Africa, health expenditure can push families into hardship and debt, which in turn reduces adherence to prolonged treatment and sustains the cycle of poverty [5].

In Australia, the *Aedes notoscriptus* mosquito is implicated with transmission, but in endemic African communities, the transmission has been associated with direct and indirect exposure to wetland areas and aquatic insects, complicating prevention strategies [71,72]. A case study in Cameroon found a decreased risk of BU by 32% and 34% with bed net use and wound care, respectively [73]. Moreover, the diagnosis of BU is frequently delayed [74]. The nonspecific and relatively painless nature of early lesions, coupled with their insidious onset, makes clinical diagnosis of early lesions difficult. While IS*2404* polymerase chain reaction is the diagnostic test of choice, access is limited in remote endemic communities, and its effectiveness is highly dependent on correct sample collection, with high false negative rates for nonulcerated lesions or incorrect swabbing techniques [75,76]. Current treatment relies on an 8-week course of rifampicin-based combination oral antibiotic therapy, which, despite being highly effective in curing the disease, presents substantial challenges [77]. These include significant toxicities, drug interactions, and the development of paradoxical reactions in approximately one in five patients, which are associated with increased tissue necrosis and delayed wound healing [78,79]. Furthermore, ulcers usually enlarge with antibiotic treatment and typically take many months to fully heal [80]. Many of these complicating factors are a likely consequence of the lipid-like *M. ulcerans* exotoxin mycolactone, which has profound and severe pleiotropic effects on host tissue [81–83]. This highlights that effective treatment alone is insufficient to address the full burden of BU, underscoring the critical need for prevention. Furthermore, a laboratory study using a murine infection model suggests that there is indeed a window during which clinical disease can be controlled by the host immune system following transmission from the environment, strengthening the case for vaccine development [84].

Currently, there is no effective licensed vaccine specifically for the prevention of *M. ulcerans* infection [85,86]. A key scientific challenge to BU vaccine R&D is the immunosuppressive nature of mycolactone [87]. In addition, the correlates of immune protection in humans are largely unknown [88]. The BCG vaccine appears to offer significant but non-durable effectiveness against BU (similar to leprosy), although early trials had several limitations [86]. In mice, recombinant BCG-vaccines, modified to express *M. ulcerans*-specific antigens, appear to be a promising advance on BCG vaccine alone [89]. More recently, a composite vaccine (Burulivac) containing two immunodominant *M. ulcerans* antigens, coupled with an adjuvant and a low dose of mycolactone, has shown sterilising immunity in a murine challenge model [90]. mRNA-based vaccines are also in development (www.baxerna.eu). However, despite the emergence of promising vaccine candidates, a major challenge now lies in translating these results into clinical trials, as no BU-specific vaccine candidate has ever reached this stage. Additionally, the sporadic and unpredictable epidemiology of BU makes large-scale field testing unfeasible. A controlled human infection model for BU is currently in development in Australia; this experimental platform holds the potential to fundamentally advance the understanding of early human immune responses and rapidly evaluate vaccine candidates, directly addressing scientific and logistical barriers [91–93].

Despite scientific breakthroughs and the potential of controlled human infection models to fast-track vaccine selection, the primary strategy by WHO for BU control focuses on early detection and antibiotic treatment, with vaccine R&D not explicitly listed as a key research priority. This absence of a clear policy focus on vaccine R&D suggests a missed opportunity and a perpetuation of a reactive, treatment-centric approach.

### Intensifying the efforts

**Fig 2:**
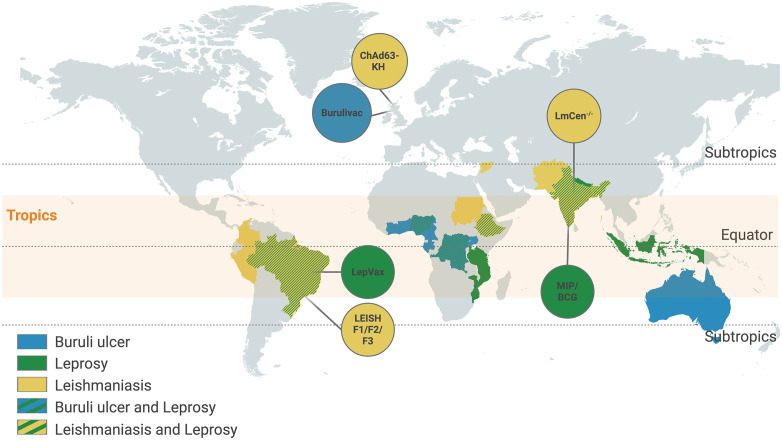
Global distribution of skin NTDs: Buruli ulcer (blue), leprosy (green), and leishmaniasis (yellow) cases, highlighting the ten countries with highest number of cases. Overlap between Buruli ulcer and leprosy is shown in blue-green stripes, and overlap between leprosy and leishmaniasis is shown in green-yellow stripes. The orange shading marks the tropics, with grey dashed lines indicating the equator and subtropics. Candidate vaccines in pre-clinical or clinical trials are also shown for leprosy (green), Buruli ulcer (blue), and leishmaniasis (yellow). Created in BioRender. Novais, F. (2026) https://BioRender.com/7tfd1o9.

For all three diseases, despite significant scientific progress in identifying vaccine candidates (Fig 2), approved human vaccines remain elusive and are not expected soon. This highlights a fundamental disconnect between market and public health need, where scientific feasibility is present, but financial incentives and regulatory pathways are not adequately aligned to support research along the technology readiness pipeline, to bring these products to market. The so-called “valley of death” between promising research and a licensed product is primarily financial and logistical barriers. To intensify our efforts and advance vaccine R&D for skin NTDs, we call for the implementation of three major pillars:

## Pillar 1: Increase in funding for skin NTD vaccine R&D

The R&D of skin NTD vaccines faces significant financial challenges because of the perception that development costs outweigh financial returns, meaning that there is no commercial incentive for pharmaceutical companies. Vaccine R&D is inherently expensive, estimated at hundreds of millions of dollars over 12–15 years, with a significant risk of failure. Hence, major global health investors have shifted their focus from NTD vaccines to diseases of emergency preparedness. Moreover, the true economic burden of NTDs is often underestimated, neglecting issues such as lost productivity, social stigma, and costs to the public health sector. This hinders the case for investment and calls for comprehensive mathematical modelling, a cost-effective tool for quantifying the true cost of skin NTDs. For policymakers and pharmaceutical companies, such models would be extremely useful when making a clear business case: while a vaccine has an initial high cost, it ultimately saves billions in avoided treatment costs and DALYs, making it a worthwhile investment. It also calls for innovative funding models and lessons, such as strengthening manufacturing and regulatory capacity in endemic regions. Donors and investors are more likely to commit resources if a sustainable and long-term impact could be expected. Therefore, linking vaccine development and technology transfer to capacity building in endemic regions would significantly reduce costs, lower risks, and ensure that funding translates into sustainable impacts. This makes the investment case for NTD vaccines not just more compelling but also more attractive to both public and private funders. The experience of COVID-19 vaccine R&D demonstrated that substantial upfront funding, advance purchase agreements, and market commitments can effectively de-risk development and incentivise manufacturers.

Priority Review Vouchers (PRVs), introduced by the U.S. FDA in 2007, incentivise the development of new drugs for certain diseases [94]. Upon FDA registration of an NTD drug, a voucher is issued that grants expedited review. These can be resold, and the estimated market value of these vouchers was estimated to be US$100 million in 2024, making them flexible, untied, financial incentives to developing drugs to tackle NTDs [95]. PRVs have been instrumental in the development of the first Chikungunya vaccine, as well as dengue vaccine, which generated a significant profit [96–98]. The European Union is actively exploring a similar, improved scheme, potentially involving the European Health Emergency Preparedness and Response Authority. However, some have pointed out flaws in and limited impact of the PRV system [99]. Similarly, the Brazilian regulatory agency, Anvisa, has a priority review system for petitions (RDC No. 204, dated December 27, 2017). Priority will be given to petitions for prior approval in clinical research processes and substantial amendments for drugs intended for neglected, emerging, or re-emerging diseases, public health emergencies.

Development Impact Bonds (DIBs) offer another novel financing model [100]. They leverage upfront private sector investment, with donors repaying capital (plus a potential return) only if clearly defined and measured development outcomes are achieved. This mechanism transfers the risk of program failure from donors to social investors, incentivising rigor and successful delivery. DIBs provide a “front-loaded” cash flow profile, ideally suited for initiating large-scale interventions like mass vaccination campaigns, which require high upfront costs to interrupt transmission, contrasting with traditional flat year-on-year funding. This approach is currently being explored for zoonotic sleeping sickness and rabies control in Uganda and is broadly applicable to most NTDs where existing disease control tools are available [101,102].

Public-Private Partnerships are also essential, as they foster collaboration between governments, academia, not-for-profit organisations, and the private sector [103,104]. For example, governmental funds such as the U.S. Centers for Disease Control and Prevention, the UK’s Foreign, Commonwealth & Development Office, and Agence Française de Développement, work extensively with pharmaceutical partners who generously donated billions of dollars in treatments. Examples are the partnerships between the Sasakawa Health Foundation and Novartis who have provided free leprosy drugs through the WHO since the mid-1990s, and the Global Health Innovative Technology Fund, which in 2023 committed ~¥150.38 million (~US$1.0 million) to advance LepVax into Phase Ib/IIa clinical evaluation led by Sasakawa Health Foundation, American Leprosy Missions, and IOC/Fiocruz in Brazil. Funding opportunities from LMICs are also important, as these countries often prioritise diseases endemic to their regions and can fund local initiatives (*e.g.*, clinical trials, vaccination campaigns, and mobile health clinics), thereby fostering regional ownership and relevance.

The success of COVID-19 vaccine R&D, driven by unprecedented public and philanthropic investment and risk-sharing, is a compelling blueprint for accelerating NTDs vaccine development. This demonstrates that the biggest obstacle for NTD vaccines is not scientific capacity but a lack of comparable financial commitment and market incentives. Advocating for an increase in NTD vaccine funding should explicitly reference the COVID-19 model as a highly effective strategy, emphasising that similar political and financial commitments can yield game-changing results. The funding strategies for NTD vaccines should aim to alter the existing market dynamics to make NTD vaccine development much more attractive to commercial entities. This will require a blend of philanthropic support, local government commitment, and innovative mechanisms that can bridge the gap to commercial viability.

## Pillar 2: Integration of NTD vaccine R&D into the preparedness and response policies by international agencies and local governments

The WHO “Ending the neglect to attain the sustainable development goals: a roadmap for NTDs 2021–2030” outlines ambitious global targets, including a 90% reduction in the number of people requiring NTD interventions, a 75% reduction in NTDs-related DALYs, and at least 100 countries eliminating one NTD by 2030 [1,2]. The roadmap strongly emphasises the need to move away from vertical disease-specific programs to integrated, cross-cutting approaches. These should focus on public health interventions such as preventive chemotherapy, vector control, veterinary health, water, sanitation, and hygiene. The WHO also published their strategic framework for integrated control and management of skin NTDs, consisting of six core interventions: early case detection, clinical diagnosis, laboratory confirmation, treatment, management of complications, and prevention [4]. Recently, at the 78th World Health Assembly in 2025, resolution WHA78.15 “Skin diseases as a global health priority” was adopted, highlighting the critical impact of these diseases [105]. While the roadmap and the framework highlight the need for these important programmatic actions and cross-cutting approaches, disappointingly, they do not explicitly prioritise vaccine R&D for leishmaniasis, leprosy, or BU, despite the transformative potential of vaccines to reduce the burden of these diseases.

The blueprint for addressing this gap comes from the tuberculosis (TB) field: in 2023, the WHO Director-General launched the TB Vaccine Accelerator Council to tackle systemic barriers in vaccine development and access [106]. This initiative encourages high-level collaboration among governments, funders, and industry to secure sustainable financing and ensure equitable distribution. Adopting a similar Accelerator Council framework for skin NTDs could enable progress where conventional strategies have failed. Under WHO leadership, a dedicated platform with skin NTD-specific working groups could prioritise vaccine candidates for leprosy and BU, leveraging advances in mycobacterial immunology from TB research. This could also include addressing the funding gaps through innovative mechanisms (*e.g.*, aggregated donor pools), similarly to strategies proposed for TB vaccine R&D. The success by the TB Council shows that overcoming neglect requires political urgency, diversified pipelines, and community-engaged R&D, all of which are highly relevant also for skin NTDs. Learning from these lessons could bridge the implementation gaps and accelerate the elimination of skin NTDs.

## Pillar 3: Fostering patient and public engagement and advocacy for skin NTD vaccine R&D

Effective patient and public engagement and policy integration are paramount for the success and sustainability of global immunisation efforts for NTDs, as they can help address vaccine hesitancy, promote adherence, and build confidence in vaccination. Community-led strategies have been shown to significantly improve health behaviours for NTDs [107]. It requires a fundamental shift towards community ownership and strengthening the underlying primary healthcare system. This involves investing in local health infrastructure, ensuring consistent supply and service delivery, and empowering local health workers and leaders to act as both implementers and advocates, laying the groundwork for lasting impact. Moreover, incorporating the patient voices in the planning of R&D process is key to aligning with clinical needs and facilitating its implementation [108]. This has been highlighted by patients for leishmaniasis vaccine development [109].

Tackling vaccine hesitancy remains a critical challenge, and was recognised as one of the top 10 global health threats in 2019 [110]. Reasons include concerns about vaccine safety and side effects, low trust in health systems in some countries, language barriers, discrimination, and cultural barriers [111–114]. For NTDs, fear or hesitancy to participate in health interventions is prominent, particularly for skin NTDs that cause lifelong disabilities, severe disfigurement, and profound social stigma. These are significant barriers to seeking health care, attending screening activities, and participating in health interventions [115]. Public engagement strategies for skin NTD diseases must therefore confront a dual challenge: overcoming general vaccine hesitancy and addressing the disease-specific stigma.

Key components of effective public engagement in health strategies for skin NTDs must include transparent and culturally relevant communication, stakeholders’ participation, trust-building with communities, and inclusion of community feedback into R&D process and health policy. Communication efforts must go beyond building vaccine confidence to actively work on de-stigmatising the disease and promoting early diagnosis and treatment as part of a comprehensive healthcare approach. Skin health campaigns that screen for multiple conditions can help reduce stigma associated with single-disease screening and potentially increase vaccine acceptance within a broader context of community well-being. Achieving this requires proactive, multi-layered strategies for improving communication, trust-building, and local ownership. For example, a key component of the LepVax project is the active involvement of members from the Brazilian organisation for people affected by leprosy, known as the “Movement to Reintegrate Persons Affected by Hansen’s Disease”. Their participation brings valuable perspectives and insights to the investigation, to improve the quality of leprosy services and increase the uptake of interventions in Brazil. By participation, they can raise awareness about leprosy and create more intervention opportunities, ultimately working towards eliminating the disease.

Moreover, training and empowering local stakeholders are crucial to any successful vaccine initiative. Community health workers should be trained in the new vaccine technologies and empowered to become advocates for vaccine confidence and equitable access, equipped with skills in digital health tools, and culturally sensitive communication, to maximise knowledge translation in rural and suburban settings. Engaging influential public figures, religious and community leaders through targeted workshops, can position them as vaccine champions. At the same time, healthcare providers need specific training on vaccine safety and efficacy, and on how to effectively disseminate that information to communities, to counter misinformation. Ultimately, public engagement must therefore extend beyond simple information campaigns, and tackle digital misinformation, foster long-term health education, ensure inclusivity, and adapt strategies that realistically capture community impact in order to build lasting vaccine confidence and sustainability.

## Conclusions and recommendations

NTD R&D relies heavily on public and philanthropic organisations and the pharmaceutical industry. However, the global health technology community has largely shifted its focus away from NTD vaccines, instead prioritising diseases of “emergency preparedness” or those requiring lower-risk technologies [116]. This is reflected in the recent G-FINDER report, which shows that the overall NTD R&D remained stagnant or declined, and that there was an increased focus on preparedness to emerging infectious diseases and mRNA technology [117].

The limited focus on vaccine R&D for leishmaniasis, leprosy, and BU in WHO Global Health policy is a manifestation of a broader systemic problem. The “neglect” inherent in “neglected tropical diseases” seemingly extends to their vaccine R&D too. The market forces do not incentivise vaccine R&D to these diseases, and global health priorities are often swayed by more immediate, high-profile threats (*e.g.*, COVID and mpox). The unintended consequence of this structural neglect means that even when promising candidates emerge from research, they struggle to secure the sustained funding and policy backing necessary for progression to licensure and widespread deployment.

Yet, vaccines for skin NTDs represent the most powerful, sustainable, and cost-effective intervention available, capable of breaking the vicious cycle of poverty and disease that traps millions of people globally. The scientific progress in identifying promising candidates for leishmaniasis (LmCen^−/−^, LEISH F1/F2/F3), leprosy (LepVax, Mw/MIP), and BU (Burulivac) demonstrates that these goals are scientifically within reach. Realising their transformative potential will now require a concerted, multi-pronged, and sustained global effort by all stakeholders involved. This includes investment in vaccine R&D through financial innovation, community empowerment and engagement, and updates in WHO response policies, more strongly advocating for vaccine R&D.

By strategically investing in NTD vaccine R&D, optimising regulatory pathways, and empowering affected communities, the global community can significantly reduce suffering, foster economic development, and advance global health equity, aligning with the ambitious targets of the WHO NTD Road Map 2021–2030 and the broader Sustainable Development Goals. The ultimate success of NTD vaccine programs is not solely about developing and deploying vaccines, but integration into a robust health ecosystem that can sustain prevention, diagnosis, and treatment of these diseases.
